# Obesogenic Environment Case Study from a Food and Nutrition Security Perspective: Hermosillo City

**DOI:** 10.3390/ijerph16030407

**Published:** 2019-01-31

**Authors:** Ana Contreras Navarro, María-Isabel Ortega Vélez

**Affiliations:** Centro de Investigación en Alimentación y Desarrollo, Hermosillo 83304, Mexico; anaconnav@gmail.com (A.C.N.)

**Keywords:** food system, obesity, diet, public health, underserved populations, measurement

## Abstract

Obesity and certain nutritional deficiencies are global health problems that emerge in systems of interdependent individual biological and historical factors and social environmental determinants of health. Nutrition security is a framework that assumes stable access to sufficient innocuous and nutritious food (i.e., food security), health care, and sanitation, and information that in conjunction allows self-care-oriented behavior for health protection. To understand the social environment of nutrition insecurity, the object of study was the food distribution and consumption system of a marginalized community in Hermosillo, Mexico. We assessed the distribution of food establishments by social marginalization level in basic geo-statistical areas and the nutrition security status of women in underserved neighborhoods. We found that in Hermosillo >90% of food establishments included for analysis (grocery stores, supermarkets, convenience stores, and beer deposits) were distributed outside of areas with high levels of social marginalization. The nutrition security assessment suggests that low intakes of fruit and vegetables and high intakes of fat and sugar may be associated with food accessibility and acceptability factors in individual decision-making processes. Future research should take into account the variability of food system environments and address the particular needs of communities in terms of food and nutrition security.

## 1. Introduction

Obesity is an emergent global health concern documented in most countries after 1975 [1]. The etiology of obesity is complex, ranging from individual biological and historical factors to social and environmental determinants of health [2]. This health condition is related to diabetes, cardiovascular disease, some cancers, obstructive sleep apnea, and osteoarthritis. According to the World Health Organization, the probability of comorbidities increases with a BMI (Body Mass Index) of ≥25.0 kg/m^2^, a measure of body composition for the adult population based on weight and height [3]. Worldwide in 2014, the prevalence of adult obesity was 13% (BMI ≥30.0 kg/m^2^), a figure that tripled in men (3.2% to 10.8%) and doubled in women (6.4% to 14.9%) over the last four decades [1]. Furthermore, some regions experience the double burden of malnutrition [4]. 

Even though undernutrition declined since 1990 (e.g., the global prevalence of chronic infant undernutrition was reduced from 39.6% to 23.8% in 22 years), certain energy and nutrient deficiencies persist [1,4]. Hunger is estimated to affect 800 million people, and 23% of children ≤5 born in localities with inadequate sources of clean water, sanitation, and food struggle with restricted growth (i.e., stunting) [4]. If these conditions are present in critical periods of human development they may further progress into the intergenerational transmission of malnutrition (e.g., maternal undernutrition increases infant vitamin A and zinc deficiencies and gestational obesity increases infant mortality) and have consequences in terms of educational achievements [5].

The energy content and nutritional quality of diets demonstrate trends in food choice interrelated in obesity and nutrient deficiencies [6,7]. In 2010, low intakes of fruits, vegetables, whole grains, nuts, and seeds had a higher contribution to the global burden of disease compared to the adult prevalence of excess weight (BMI ≥ 25.0 kg/m^2^) [7]. Studies of dietary patterns in Canada, the United States, and Brazil suggest a global shift towards ultra-processed food products that result in reduced dietary contents of fiber, protein, and vitamins (e.g., vitamins D and E) and minerals (e.g., magnesium) and increased contents of fat and sugar [8,9,10]. Another key behavioral risk factor involved in malnutrition is physical inactivity, most prevalent in cities [7,11]. 

In response, public nutrition and health research produced ecological models of obesity [12,13,14,15]. For example, in 1999 two articles used the terms *obesogenic environment* and *toxic environment* to describe characteristics in cities and patterns in behaviors that explain the emergence of the obesity epidemic [12,13]. The complex nature of these co-evolving macro- and microstructures is captured by principles and tools from systems science that can be applied to community-based studies of food and nutrition security [16,17,18,19]. Food and nutrition security “exist when all people, at all times, have physical and economic access to sufficient, safe and nutritious food to meet their dietary needs and food preferences for an active and healthy life” [20]. This scenario is uncommon in practice because it requires coordination in the activities of agriculture, food, health, and environmental systems that shape individual decision-making processes and nutrition outcomes [16]. 

According to the U.S. Institute of Medicine, a food system, commonly represented as a supply chain, is configured by interdependent and dynamic processes (selection, production, distribution, consumption, waste), heterogeneous autonomous agents (consumers, farmers, restaurant-owners, companies, NGOs, bacteria, fungi), and shared structures between agents (social networks, cities, schools, norms, media) [18]. Given the broad domains and dimensions of food systems, in this case study we focus on distribution and consumption processes of an underserved consumer population to understand the social environment of food and nutrition insecurity. In the following sections, we describe the framework that guides this research in consideration to an identified gap in food environment studies, followed by a literature review of food environment research in Mexico and the description of the case study in Hermosillo City.

### 1.1. A Tool to Assess Food Systems for Nutrition Security

**Figure 1:**
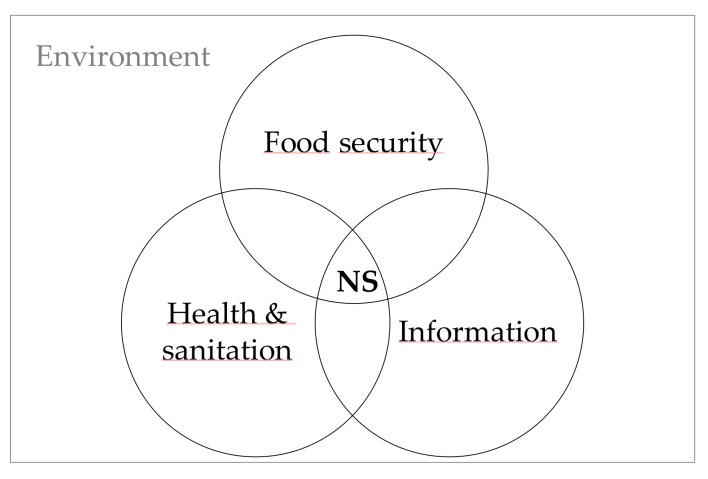
Conceptual framework of nutrition security (NS) adapted from Sanchez-Griñan (1998).

Nutrition security is a framework based on community studies aimed to identify the determinants of nutrition [21,22]. It assumes stable access to sufficient innocuous and nutritious food (i.e., food security), health care and sanitation, and information that in conjunction allow self-care-oriented behavior for health protection (Figure 1). It serves as an assessment guide that goes beyond the individual level extending to structural (physical and socioeconomic) factors. In 2013, FAO enlisted 30 indicators of food and health supplies that are interrelated with nutritional processes and outcomes [23] (p. 16). These cover broad contextual factors including availability, accessibility, affordability and stability of food, which are key concepts in food environment frameworks [14,15,24,25].

The food environment refers to “the food available to people in their surroundings as they go about in their daily lives and the nutritional quality, safety, price, convenience, labelling and promotion of these foods” [25]. Researchers in the field are increasingly using geographic information systems (GIS) to characterize the distribution and type of food stores and restaurants in specified areas [24,26]. However, assumptions of these models may be faulty because proximity to stores “does not account for travel patterns, taste preferences, social norms about where to procure food, or ability to afford foods” [26]. This was reported by Lytle and Sokol who revised 432 articles on quantitative measures of the food environment published between 2007 and 2015. A useful comprehensive review on qualitative studies captures this existing gap [27].

The limitation of geographical analysis in environmental research and health behavior leads to other proposals focused on individual activity patterns aimed to find key interactions between structural features and consumer food choice [28,29]. For example, in Denmark, Lyseen et al. tracked 187 adults aged 16–23 using a GPS device for a week to test the validity of generalizations based on studies using “neighborhood” or district areas made available by state agencies. The authors discuss that even if people share community, school or workplace, individual behavior is diverse: ~87% of the participants traveled outside their home neighborhood to attend schools (where exposure to supermarkets and fast food outlets increased) and 15% of mobility occurred outside of measured home and school neighborhoods [28]. Furthermore, each environment is influenced by unique geographic, biophysical, cultural and economic systems [18] and, as such, represents the space linking particular food systems and diets [25].

From a systems science perspective, an innovative computational technique that can be used to generate simulations of social processes involved in the dynamics of malnutrition is Agent-Based Modeling (ABM) [16]. Agent-based models simulate both behavior and contextual factors to understand/predict patterns observable in complex social systems [16,17,30]. Even though the intent is not to construct an agent-based model, we make use of an organization tool developed within the field of ABM, the PARTE framework, to identify food system structure and activity in relation to nutrition security [17]. An initial step is the specification of Properties, Actions and Rules of agents and Time and Environment of context relevant to the system under study. Properties refer to agent characteristics, such as age and health status; actions in food systems would include food selection, production, distribution and consumption; rules are consumer strategies and belief systems involved in food decision-making processes. Time and Environment depend on research questions and experiment design.

### 1.2. Food Environment Case Study: Hermosillo City

Mexico is challenged by the double burden of malnutrition. On the one hand, overweight and obesity are estimated to affect 70% of adults aged ≥20 and 34% of school-age children [31,32]. In agreement with global trends, between 1988 and 2006 the prevalence of obesity in women increased from 9.5% to 34% [31]. It is also estimated that 3.5 million women and 2.8 million men have diabetes [33]. In fact, as of 14 November 2016, obesity and diabetes called for a nationwide epidemiological emergency [34]. On the other hand, 13% of children ≤5 are stunted, a prominent problem in rural and indigenous localities where this estimate rises to 27% [31]. 

Maize, wheat, rice, and other cereals are basic foods that provide ~32% of the total energy in the average diet. However, 26% is attributed to the consumption of sweet bread, deep-fried corn snacks, sugary drinks and other non-essential foods, while 6% comes from vegetables and fruits [35]. This type of dietary pattern contains less fiber, vitamins, minerals and phytochemicals involved in biological systems that are protective for health [9,10,36]. Two fiscal policies directed to shape the consumption of energy-dense snacks (≥275/100 g, 8% tax) and sugar-sweetened beverages ($0.05 USD per liter, 10% tax) were implemented in 2014 [37], a strategy that targets the consumption of widely available foods, independent of region (urban/rural localities) or setting (restaurants/street vendors). 

Over the last 70 years, the food distribution system transitioned from “traditional” markets to the expansion of supermarkets, convenience stores, fast food chains and specialty stores. Changes in the retail environment occurred especially after 1980 [38,39]. An illustrative example is the integration between multinational company Walmart and Mexican Grupo Cifra in 1991. By 2012, the commercial enterprise operated 1140 stores in 230 localities, covering 60% of consumer demand in urban areas [39]. Previous research has highlighted that supermarkets, convenience stores, and fast food outlets cluster around main avenues or federal roads and shopping centers, usually not in high-poverty areas, leading to disparities in access to these establishments in metropolitan areas [40]. In contrast, the food environment for underserved populations is characterized by greater availability of corner stores, offering a stable supply of ultra-processed foods/snacks and specialty outlets selling traditional products [41].

Several studies suggest that limited access to fruits and vegetables, stable product availability and food acceptability are important system drivers of consumption patterns for low-income populations [41,42,43,44]. In Mazatlán, Sinaloa, low-income neighborhoods were described as food swamps compared to high-income areas because people are exposed to more ultra-processed foods [41]. In Cuernavaca, Morelos and Guadalajara, Jalisco, the food environment around public schools was characterized by higher availability of street vendors offering unhealthy food compared to the food environment around private schools [42]. In Tijuana, Baja, California, a dietary assessment for 2345 women, showed variance in dietary patterns in relation to income and education. In low-income women, a diet based on maize, rice, and beans was more prevalent, while women in the high-income group tended to eat more vegetables, fruits, and whole grains [43]. The latter results are similar to a study that used nationally representative data from 2006 in which patterns based on maize and beans correlated with lower BMI in adults, while the consumption of vegetables was found to be more common in women of older age [44].

**Figure 2:**
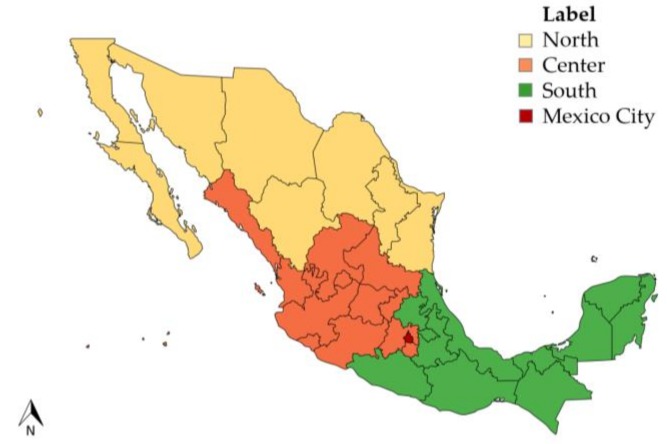
Mexico map by region (mapchart.net©).

Furthermore, there are regional differences in nutrition and dietary patterns across the country. In northern states (Baja California Norte, Baja California Sur, Chihuahua, Coahuila, Durango, Nuevo León, Sonora, Tamaulipas) the highest prevalence of obesity in adults (37.8%) is reported compared to central (29.4%) and southern (34.7%) regions [32] (Figure 2). The highest energy intakes of sugar-sweetened beverages and animal products and the lowest intakes of fruits and vegetables are also reported in northern states [32,35]. In contrast, some southern states experience high social marginalization and distinct nutritional outcomes (stunting) [31].

In particular, in the northern state of Sonora, known for agricultural region Yaqui Valley, the home of the Green Revolution for wheat [45], it was previously reported that the prevalence of obesity in women who migrate for work in agricultural fields, from Oaxaca, Veracruz, and Guerrero, is greater after four years of residence, compared to women with less time in the region [46]. Therefore, given the high prevalence of obesity and the differences in dietary patterns, it represents an example of an obesogenic environment for transition populations (migrants and recently urbanized). The case study is based in Hermosillo City, which is located 179 miles from the USA–Mexico border and has a population of 812,229 people. Estimated life expectancy is 72.7 years for men and 78.6 years for women (global estimates are 69.8 and 74.2, respectively) [47] and the main causes of disability and premature death are ischemic heart disease, diabetes, and chronic kidney disease [48]. 

## 2. Materials and Methods

### 2.1. General Design and Procedures

In this case study, datasets from Mexican agency INEGI (Instituto Nacional de Estadística, Geografía e Informática) were used to examine the distribution of food establishments in Hermosillo [49]. We report a qualitative and spatial analysis of regional and national food networks by marginalization degree assigned to basic geo-statistical areas (AGEB). We used the social marginalization index developed by CONAPO (Consejo Nacional de Población). It contains nine items of social deprivation in relation to housing, education, social security, income, and population density [50]. An AGEB is the second smallest census unit composed of 1–50 neighborhood blocks each containing aggregated data for 190 socio-demographic indicators. A total of 410 AGEB classified in one of five degrees of social marginalization: 38% very low, 20.1% low, 16.8% medium, 3.6%, high, 3.2% very high and 17.3% had no available information.

From April to June 2018, fieldwork followed in two neighborhoods with a very high degree of social marginalization to assess measures of nutrition security: dietary patterns, nutrition status, household food security and perceived food environment and behavior. In-person questionnaires and anthropometric data were collected by trained personnel. In accordance with the Declaration of Helsinki, the study protocol was approved by the institutional review board at Centro de Investigación en Alimentación y Desarrollo (CE/004/2018) and the informed consent procedure followed the Mexican norm for human subjects research (NOM-012-SSA3-2012).

### 2.2. Distribution of Food Establishments

Food establishment information was obtained from INEGI geo-statistical system DENUE (Directorio Estadístico Nacional de Unidades Económicas), last updated March 2018 [49]. From a database of 9590 registers of economic units in Hermosillo (covering information on identification, class of economic activity, address and spatial coordinates), commercial food networks were selected for analysis (*n* = 760). Specifically, we included grocery stores from regional food chains and supermarkets, convenience stores and beer stores (i.e., deposits, franchise beer outlets similar to convenience stores in that venues have private lots for customers to park, pick and go) from national food chains. Food chains operated nationwide were previously identified in research on the evolution of the commercial food distribution system in Mexico [39]. Spatial analysis was done in QGIS 3.2.3.

### 2.3. Participants

Women present the highest prevalence of obesity among the total population in the country [31] and a higher prevalence of inadequate intake of vitamins (vitamins D, A, folate, B complex), compared to men [51]. Women’s food choices within the household may reflect their partner’s food preferences [52,53] and influence children’s diets [54]. Previous research also provides evidence for an association between food insecurity and obesity in women in Mexico and elsewhere [55,56]. Therefore, during fieldwork in two neighborhoods (among a set of 13 AGEB with a very high degree of marginalization), we asked women aged 18–49 to participate in the study if they had lived in the neighborhood for ≥4 years to control time of exposure to the local food environment between participants. 

### 2.4. Fieldwork Materials

In-person questionnaires included (1) a single 24-hour dietary recall, (2) a household food security scale, and (3) a perceived food environment and behavior survey, described below. 

(1) The 24-hour dietary recall (24-HDR) technique involved an interviewer asking the participant to describe all food and drink consumed in the 24-hour period before the interview, including portion size (assisted by food models) and recipes (when prepared at home) following the five-step multi-pass method [57]. Portion size was treated as grams and nutrient composition of each food was collected from a regional food database [58]. 

(2) We used a household food security scale developed and validated for the northern region of Mexico, similar in format to the Household Food Insecurity Access Scale and the Latin American and Caribbean Food Security Scale (ELCSA), to capture household and individual access to food, as well as emotional perceptions streaming from food deprivation (food anxiety and lack of choice) [59,60]. The number of affirmative answers (15 Yes/No questions) was used to categorize households in food security or food insecurity. Food insecurity progresses from perceived stress and anxiety related to poor diet quality affecting preferences and dietary diversity (low), to the reduction of diet quality and quantity among household members (moderate), to physical symptoms of hunger in extreme poverty conditions (severe). 

(3) An adaptation of the Perceived Nutrition Environment Measures Survey was used to study the perception of the local food environment and food shopping behavior. Glanz and colleagues designed four instruments to measure structural factors in defined scenarios (restaurants, stores, corner stores, vending machines) and a non-observational survey to capture the individual’s perspective on the food environment (NEMS-P) [14,61]. We used a shorter version of NEMS-P focused on food shopping behavior. Questions included for analysis were (i) Who shops for food consumed at home? (ii) Where do you shop for food consumed at home? (iii) How often do you visit each of these stores? 

Anthropometry was measured to estimate body composition and nutrition status. The equipment used was a standing electronic scale (capacity 0–200 kg ± 0.05 kg), a portable stadiometer (capacity 30–205 cm ± 1 mm) and a measuring tape (0–205 cm ± 1 mm). During measurement, participants wore light clothes, an examiner helped position the participant and an assistant recorded weight, height and waist circumference (WC). Nutrition status was assessed estimating BMI (kg/m^2^) in reference to WHO: low weight <18.5, normal weight >18.49 and <25, overweight >24.9 and <30, obesity >29.9 [62]. Abdominal obesity was defined as WC >79 cm in accordance with the Mexican norm NOM-043-SSA2-2012.

Other variables collected using a sociodemographic questionnaire included age, education, employment, housing and kitchen conditions (i.e., Do you own a refrigerator?), access to social security, time of residence in neighborhood, and participation on food assistance programs.

### 2.5. Fieldwork Data Analysis

Descriptive individual and household characteristics were analyzed in NCSS: Statistical Software 12.0.9. To analyze dietary patterns, total energy, protein, fat, carbohydrate and fiber were estimated. A list of 210 food items were categorized in one of 10 food groups, using the macronutrient content informed by an educational guideline for Mexican nutritionists (Sistema Mexicano de Alimentos Equivalentes) [63]. Thirteen items reported as recipes were broken down into basic ingredients. Food groups were: (i) vegetables; (ii) fruits; (iii) cereals and tubers; (iv) legumes; (v) animal products; (vi) milk and dairy; (vii) oils and fats; (viii) sugar and sugary drinks; (ix) high added fat/sugar products; (x) ingredients with no energy content. The contribution of each group to total dietary energy is reported. The analysis followed procedures described in previous research on Mexican dietary patterns [64,35].

## 3. Results and Discussion

### 3.1. Distribution of Food Establishments in Hermosillo City

**Figure 3:**
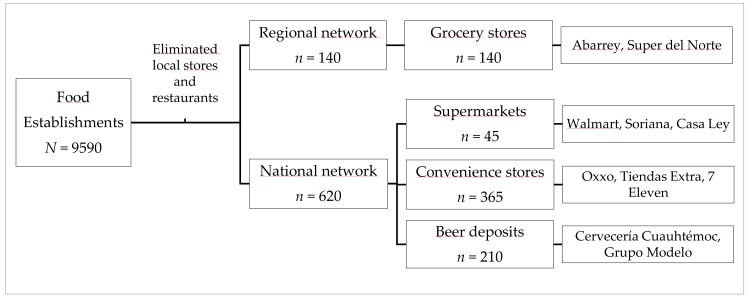
Categories of food establishments included for analysis (*n* = 760). Source: Own elaboration (2018), data obtained from INEGI DENUE.

Figure 3 showed the categories of food establishments included for analysis. The selection of grocery stores, supermarkets, convenience stores and beer deposits, from a database that included restaurants, corner stores, specialty stores, informal food stands, and other types of economic units registered in INEGI DENUE, was based on two criteria that facilitated the identification of establishments belonging to large commercial networks, namely business name and class of economic activity. 

Out of the 760 food establishments, 90% of grocery stores, 93% of supermarkets, 92% of convenience stores, and 98% of beer deposits were distributed in areas with low or medium levels of social marginalization (Table 1). None of these establishments were found in the most marginalized areas, whereas 54% concentrated in the least marginalized areas. In areas with a high degree of marginalization, we identified one grocery store, four convenience stores, and two beer deposits. This may be explained by the population density in areas with very high and high levels of marginalization, which represented 5.2% of the total population in the city in 2010 (37,368 people) [50]. There was also a higher frequency of regional grocery stores (*n* = 140) compared to supermarkets operated by national chains Walmart, Soriana and Casa Ley (*n* = 45), even when the latter have expanded their consumer markets since 2008 with the introduction of low-price neighborhood stores in Mexico (e.g., Walmart Bodega Aurrerá Express) [39]. 

**Figure 4:**
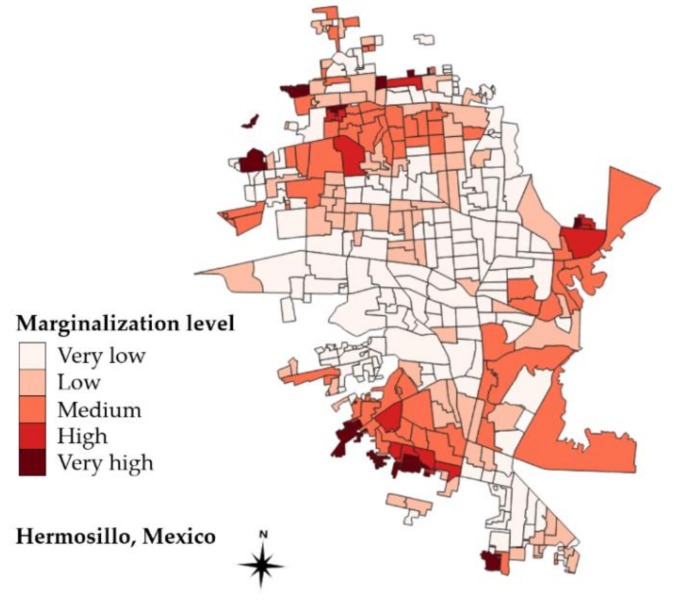
Basic geo-statistical areas by social marginalization level in Hermosillo City (*n* = 410 AGEB).

**Figure 5:**
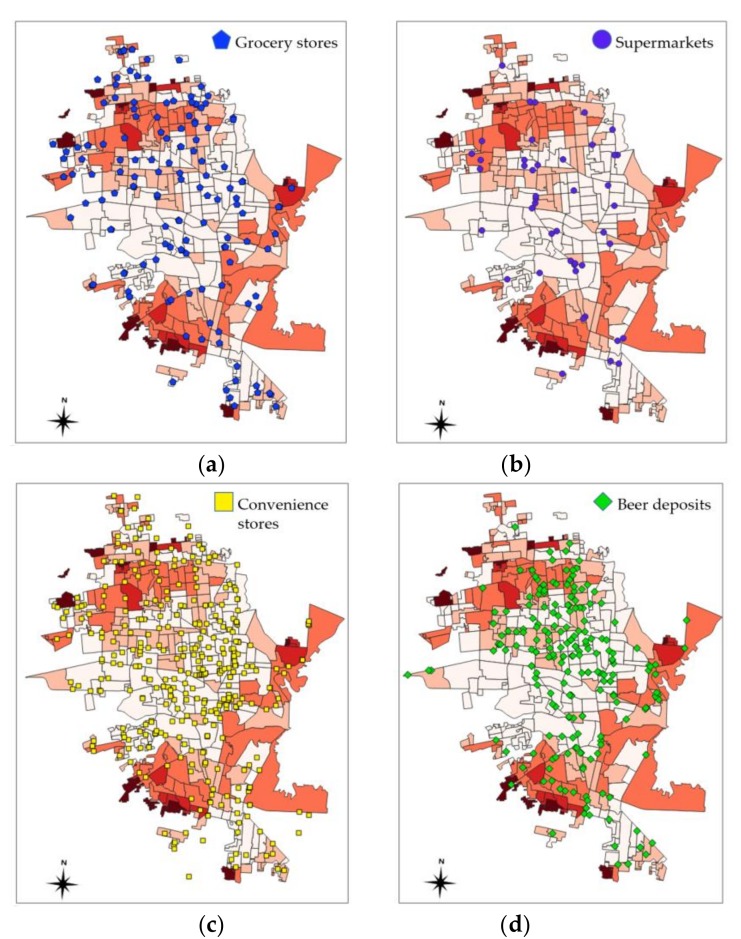
Spatial distribution of food establishments in Hermosillo (*n* = 760): (**a**) grocery stores, (**b**) supermarkets, (**c**) convenience stores, and (**d**) beer deposits.

Figure 4 and Figure 5 showed the distribution of social marginalization and a qualitative spatial analysis of commercial food establishments in Hermosillo. Patterns of segregation can be observed. All of the underserved neighborhoods were found in the periphery of the city. As discussed above, the analyzed food stores were visibly concentrated in the least marginalized areas. Previous research in northeastern city Monterrey, Nuevo Leon, reported a high concentration of fast food restaurants, supermarkets and convenience stores in main avenues, federal roads, and inside of shopping centers established outside of high-poverty polygons [40]. 

Other studies provide evidence for disparities in food distribution systems within and between regions [65,66]. A countrywide study in New Zealand documented a higher availability of fast food, take out restaurants, convenience stores and supermarkets in high-poverty zones compared to low-poverty zones [65]. In the United States, a retrospective longitudinal analysis of the food environment in Massachusetts reported that unequal access to fast food restaurants remained stable during the last four decades in low-income neighborhoods, in comparison to high-income areas in which access to fast food increased progressively throughout the years [66]. Thus, our results further the discussion on the characteristic heterogeneity of food systems, previously described as complex and adaptive [18] by providing explanatory inputs for the contradictory results between analytical studies based on food environment and nutrition measures [24,26]. 

An example of contradictory analytical results in current food environment studies was reported by Barrera et al. [42]. In their study of food establishments around private and public elementary schools and the nutrition status of children attending those schools, the authors detected a statistical positive relationship between number of mobile food vendors and children’s BMI. Based on this association, a higher BMI in children exposed to more mobile vendors outside of schools was expected. However, although the number of mobile vendors was statistically higher around public schools, there was no statistical difference between the children’s BMI attending public and private schools. As discussed by Lytle and Sokol, the identification of key mediating individual and social factors within proximate settings is needed to improve construct validity between food environment measures and health outcomes [26]. In this context, the recognition of variability in environmental exposures (e.g., mobile vendors are not fixed features of the food environment) and activity patterns has methodological implications [17]. 

### 3.2. Nutrition Security of Women in Underserved Neighborhoods

**Figure 6:**
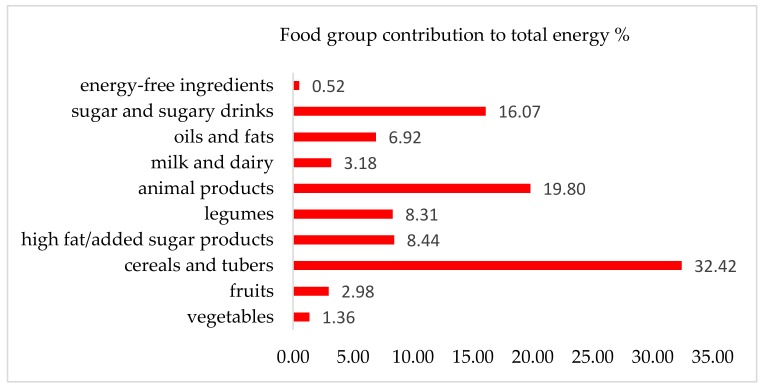
Food group contribution to total dietary energy (*n* = 67 participants).

In the assessment of dietary patterns, estimated average total energy intake was 1320.1 ± 724.1 kcal/participant. Energy derived from the mean intake of 45.5 ± 33.8 g of protein, 48.3 ± 33.4 g of fat, and 181.8 ± 97.7 g of carbohydrates, while fiber consumption was 18.3 ± 14.9 g. In reference to the nutrition guidelines on the macronutrient composition of diets for healthy adults, the contribution of fat to total energy is slightly high at 31.7% (adequate intake is 25–30%), while carbohydrate and protein are within the recommended intervals at 55.2% and 13.1%, respectively (data not shown) [67]. Tortillas, bread, potatoes, rice and other cereals contributed 32.42% of total energy derived from foods, followed by animal products (mainly beef, cheese, chicken, and pork) (19.80%), sugar and sugary beverages (16.07%), ultra-processed foods with high added fat/sugar (8.44%), beans, lentils, and chickpeas (8.31%), oils and fats (6.92%), milk and yogurt (3.18%), fruits (2.98%), and vegetables (1.36%) (Figure 6) (see the Appendix for examples of foods in each category). 

The dietary analysis showed a high consumption of non-essential foods. The combined proportion of sugar, sugar-sweetened beverages, and high added fat and sugar products (e.g., deep-fried corn snacks and cookies) contributed 24.5% of total energy. In addition, the foods most frequently reported were flour tortillas, maize tortillas, sugar-sweetened beverages, beans, and table sugar. According to Bojorquez et al., the foods in this type of dietary pattern provide high energy at low economic cost for low-income women in Tijuana, Baja California [43]. However, in 2006 this diet was more prevalent in rural areas compared to urban areas in Mexico [44]. The global increases in prices of vegetables and fruits during the last two decades may play a role in accessibility perception to these specific foods and in the overall nutritional quality of diets for marginalized groups in Hermosillo [68]. Furthermore, the protein intake suggests a sharp decrease from 72 g/d reported in the 1998 Sonoran State Food Basket study [69]. In this respect, a dietary analysis based on one 24-HDR does not provide the whole picture of habitual food practices [57]. 

In agreement with the dietary analysis, other studies show that fiber consumption of women in Mexico is lower than the recommendation set at 25 g/d [67]. In 2012, average fiber intake in low-income women was 24 g/d and 19 g/d in urban areas [70]. We emphasize fiber consumption because it reflects the quality of cereals in the diet, and also because the absorption of phenolic compounds proven to be beneficial for gut microbiota health depends on the structural matrix of whole grains. In other words, the fiber content in cereals may affect the supply of antioxidants necessary for basic functions in the digestive system [71].

The nutrition assessment based on WC indicates that 20.7% of the participants are at lower risk of developing disease related to body visceral adipose tissue (Table 2). However, 79.3% have abdominal obesity, a proportion comparable to the national prevalence of overweight and obesity based on BMI reported at 72.2% in 2016 [32]. In adult women, the likelihood of developing diabetes and cardiovascular disease increases with a BMI ≥ 25.0 kg/m^2^. However, 58% of the participants have access to limited primary health care. 

In addition, 18% of the participants perceived having an adequate access to the food supply, while 82.1% perceived food insecurity, an indicator of the availability, accessibility, and acceptability of foods consumed at home (Table 3). In contrast with the body composition results, 17.9% said members within the household experienced hunger some time during the month before the interview. An equal number of participants reported not having access to sewer services and 9.0% do not have a refrigerator at home. Both of these facts are useful to comprehend the socioeconomic environments underlying the common experience of hunger in scenarios in which a high prevalence of women have obesity. 

The results of the perceived food environment and behavior survey indicate that physical access to supermarkets, grocery stores, convenience stores, and beer stores affects consumer behavior because 49% reported walking for grocery shopping (which affects the frequency of food shopping and may limit the quantity of food carried back home) [41] (Table 3). When asked about the frequency of food shopping, 44.8% said they visited supermarkets or grocery stores once a week and 64% visited neighborhood corner stores 4–7 days a week (Table 4). These types of food establishments offer a stable and diverse supply of snack foods, sugar-sweetened beverages, and other non-essential ultra-processed foods [41,53,72]. Seemingly, these may be common consumption strategies in underserved populations, given that 73% of the participants mentioned visiting 3–4 stores for grocery shopping. Qualitative studies of the food environment indicate that consumers make purchases in multiple stores in consideration of perceived food prices and preferences [27]. 

Since most of the participants reported shopping in neighborhood corner stores frequently, more than in commercial food retailers, food access, in terms of distance and transportation, is an important environmental factor involved in food decision-making processes of marginalized populations. The walkability factor was also identified in a review by Pitt et al. [27]. Based on this results we also suggest that recognizing the multi-sectorial, multi-scale nature of local food systems can lead to tailored community programs that target specific community needs involving consumers, store owners, policy-makers, and local farmers, as has been the case for the national school feeding program in Brazil [73]. 

### 3.3. Limitations and Future Directions

This study had several limitations. Specifically, food establishment data obtained from INEGI were not physically validated. Field observations indicate that the location/operation of economic units may not be up-to-date in geo-statistical system DENUE. We suggest corroborating the data with other datasets or direct field observations. In attention to this problem, a research strategy used in a study by Sushil et al. was to validate food establishment data with the Google Geocoding application and by directly contacting a subsample of retailers to verify the address and physical location [65]. Future studies may benefit from the integration of food and nutrition security frameworks with systems science tools (the PARTE framework), as it provides useful insights for research designs on food environments and nutrition outcomes. The next step in the construction of a model of a food system would be to consider field methods that capture rules involved in individual decision-making processes. We also suggest taking a positive deviance approach, in which the case study focus are outliers (e.g., households with food security), to identify consumer strategies that can be replicated by other community members.

## 4. Conclusions

In Hermosillo, the majority of grocery stores, supermarkets, convenience stores, and beer deposits operated by commercial regional and national food networks were distributed in areas with low marginalization levels. The variability in the food supply may be interrelated with accessibility and acceptability factors that explain dietary patterns high in fat and sugar and low in fruits, vegetables, and other antioxidant- and fiber-rich food sources. An overview of the food environment emphasizes the need to identify key features susceptible to change and with the capacity to influence populations on a massive level without producing unintended effects, beyond the unique fiscal policies already implemented in Mexico and other countries. Furthermore, the turn of public nutrition towards food labeling and other information-based programs may be overlooking the needs of underserved populations. In this context, unequal access to food and health supplies underlying the obesity epidemic and nutritional deficiencies should be accounted for in nutrition security research.

## Figures and Tables

**Table 1:** Distribution of 760 food establishments by marginalization level (*n* = 410 AGEB). Source: Own elaboration (2018), data obtained from INEGI DENUE.

Network Type	Regional	National		
Marginalization level in AGEB (*n*)	Grocery stores *n* (%)	Supermarkets *n* (%)	Convenience stores *n* (%)	Beer deposits *n* (%)
Very low (156)	62 (44.3)	28 (62.2)	225 (61.6)	94 (44.8)
Low (86)	38 (27.1)	10 (22.2)	69 (18.9)	70 (33.3)
Medium (69)	27 (19.3)	4 (8.9)	43 (11.8)	42 (20)
High (15)	1 (0.7)	0 (0)	4 (1.1)	2 (0.9)
Very high (13)	0 (0)	0 (0)	0 (0)	0 (0)
Unclassified (71)	12 (8.6)	3 (6.7)	24 (6.6)	2 (0.9)
Total	140	45	365	210

**Table 2:** Individual-level properties (*n* = 67 participants).

	Mean (SD, Min–Max) or %
Age (years)	34.9 (8.36, 18–49)
Education (years)	8.6 (2.64, 0–15)
Employment Yes	26.9%
Access to health care* Yes	95.5%
Obesity (BMI > 29.9 kg/m^2^)	39.3%
Abdominal obesity (WC > 79.9 cm)	79.3%

Sample size *n* = 67 except for BMI (*n* = 61) and WC (*n* = 58); * 58% have access to Seguro Popular, which includes limited primary health care.

**Table 3:** Household-level properties (*n* = 67 households).

	%
Food insecurity No (Food Security) Low Moderate Severe	17.9% 25.4% 38.8% 17.9%
Access to sewer services Yes	82.1%
Access to refrigerator Yes	91.0%
Owns at least one car Yes	53.7%
Receives food assistance No Yes (Prospera) Other	62.7% 25.4% 11.9%

**Table 4:** Food behaviors measured with NEMS-P (*n* = 67 participants).

	*n* (%)
Takes care for food shopping and preparation in household No Yes	10 (15%) 57 (85%)
Number of stores visited for grocery shopping 1–2 3–4 >4	11 (16.4%) 49 (73.1%) 7 (10.4%)

## Appendix A. Obesogenic Environment Case Study from a Food and Nutrition Security Perspective: Hermosillo City

**Table A1:** Food groups by content.

Food Group	Contains
Vegetables	Celery, broccoli, zucchini, onion, canned mushrooms, Chile, green beans, canned corn, lettuce, nopales, pureed tomato, cabbage, salsa, carrots
Fruits	Strawberries, orange juice, lemon, mango, orange, papaya, pear, banana, watermelon
Cereals and tubers	Rice, oatmeal, potatoes, wheat and corn cereals, tortillas from corn and wheat flour, corn tortillas, wheat tortillas, crackers, home-made popcorn, bread, pasta
Legumes	Beans, chickpeas, lentils
Animal products	Beef, chicken, pork, eggs, cheese, ham, sausausage
Milk and dairy	Milk, yogurt
Oils and fats	Canola oil, olive oil, soy oil, avocado, butter, cream, bacon, almonds, chorizo, melted cheese
Sugar and sugary drinks	Sugar, hard candy, sodas, packaged fruit juice, packaged teas, gelatin, horchata, lemonade, catsup, chocolate milk
High fat and/or added sugar products	Deep-fried corn snacks, cookies, pan dulce (sweet bread), tamales, chocolate, ice cream
Ingredients with no energy content	Coffee, chicken or beef broth, sweeteners, herbs
